# EuroTau: towing scientists to tau without tautology

**DOI:** 10.1186/s40478-017-0491-z

**Published:** 2017-11-29

**Authors:** Amrit Mudher, Jean-Pierre Brion, Jesus Avila, Miguel Medina, Luc Buée

**Affiliations:** 1 University of Southampton Highfield Campus, Faculty of Natural and Environmental Sciences, Center for Biological Sciences, Southampton, UK; 2 Laboratory of Histology, Neuroanatomy and Neuropathology, Faculty of Medicine, Université Libre de Bruxelles, ULB Neuroscience Institute, Brussels, Belgium; 3 Centro de Biología Molecular “Severo Ochoa” CSIC-UAM, Universidad Autónoma de Madrid,C/ Nicolás Cabrera 1, 28049, Madrid, Spain; 4 CIBERNED, Network Center for Biomedical Research in Neurodegenerative Diseases,Madrid, Spain; CIEN Foundation, Queen Sofia Foundation Alzheimer Center, Madrid, Spain; 5 Univ. Lille, Inserm, CHU-Lille, UMR-S 1172, LabEx DISTALZ, 59000 Lille, France; 6 Inserm U1172, Univ. Lille, Fac. Medecine, Place de Verdun, 59045 Lille cedex, France

What a change in situation... A few years ago, at the height of the amyloid cascade, tau biologists, (so called Tauists) were virtually invisible in Alzheimer’s disease conferences, which were occupied by amyloid biologists (so called baptists). Currently, sessions dedicated to tau and Tauopathies are increasing in several congresses on neurodegenerative diseases, including AAIC and AD/PD. Interest in tau biology is so great that a Tau consortium, set up especially to provide a forum for this area of research, has been created in the US. In Europe, tau biologists have gathered in tau-focused meetings organized in Cambridge, UK (2010, 2012), Madrid, Spain (2013) and more recently in Lille, France (2017). The microtubule-associated tau protein is not a new protein, it was discovered in 1975, and has featured in the game of neurodegenerative disorders since 1985. Tau is now the “Figura”, and the renewed interest in this protein leads one to ask “Why such interest in tau proteins and why now?”.

There are many reasons for this burgeoning interest. Firstly the fact that most amyloid-centred therapies for Alzheimer’s disease (AD) and related disorders have demonstrated very modest, symptomatic efficacy, leaving an unmet medical need for new, more effective therapies. While drug development efforts in the last two decades have primarily focused on the amyloid cascade hypothesis, with disappointing results so far, tau-based strategies have, until recently, received little attention. This is despite the presence of extensive tau pathology, which is central not just to AD but is a key component of several other neurodegenerative diseases collectively called “Tauopathies”. Thus, focusing on tau as a drug target can have a profound bearing on disease-modification for several neurodegenerative conditions facing our ageing society today.

Secondly, multiple facets of tau biology, and therefore manifold potential implications for its role in Tauopathies, have emerged recently. Several laboratories world-wide made the seminal discovery that tau is the main component of the neurofibrillary tangles (NFT) found in AD patients more than thirty years ago, but since then, evidence has accumulated showing that posttranslational modifications such as acetylation, glycosylation, phosphorylation and truncation, among others [10, 14, 18] are pivotal in regulating tau functions.

Thirdly, the discovery of some families with highly penetrant, dominant mutations within the tau gene causing fronto-temporal lobar degeneration [8] demonstrated that tau dysfunction, including its alternative splicing is sufficient to cause neurodegeneration and clinical dementia [1, 8, 14, 15]. Whilst it is still not clear how the mutations in the tau gene cause neurodegeneration, the overall effect of these mutations is predicted to be an increase in the rate of tau aggregation and eventually the formation of insoluble tau inclusions.

As a result of this growing interest in tau biology, new hypotheses on the physiological and pathological role of tau are growing. It is no longer believed to be simply a microtubule-associated protein (MAP) [10] with recent advances in our understanding of tau’s cellular functions revealing functions beyond its classical role as a MAP. This has provided novel insights into its causative role in neurodegeneration. Such functions include neuronal polarization, axonogenesis, interactions with the plasma membrane and scaffold proteins, signal transduction, cell cycle, DNA/RNA protection, determination of dendritic spine density, and regulation of normal synaptic function [4, 11, 17]. Some of these are actively being pursued at present [12], thus broadening our range of potential therapeutic tools to treat AD and other tauopathies. Collectively, the recognition of tau as a key player in the pathobiology of human neurodegenerative diseases has driven substantial efforts to understand its biological and pathological functions.

The spread of tau pathology through the brain of tauopathy patients has been the subject of recent research because of the appearance of Aβ deposits and tau aggregates in the human brain as a function of age suggest that tau inclusions appear earlier than amyloid β plaques [2, 6]. Tau aggregates in the locus coeruleus are seen in young individuals and the typical AD associated tau pathology manifests in the entorhinal cortex from where it spreads to other brain regions.This differential distribution underlies the Braak staging for tau pathology in AD [2], but similar stereotypical spatiotemporal spreading of tau inclusions has also been described in other tauopathies such as argyrophilic grain disease [13]. Traditionally, this spatio-temporal spread of tau pathology through brain regions was believed to occur in a cell autonomous manner with the spread being determined by differential susceptibility of tissues affected. Numerous reports now challenge this view and suggest that tau pathology propagates from cell to cell and this underpins its spread through anatomically connected brain regions [3, 5]. Furthermore, evidence is emerging that these tau aggregates can adopt distinct conformations or ‘strains’ with remarkable differences in their structural and phenotypic traits [9]. This idea has been denoted the “prion-like” hypothesis and it predicts transmissibility and seeding mechanisms of many amyloidogenic proteins including tau. This idea describes spread of tau pathology but does not necessarily explain spread of neurodegeneration because it is not yet clear how and if the two are related in Tauopathies. Moreoever, there is of course the possibility that, some tau assemblies in specific conformations may not be toxic, and may in fact be inert or even neuro-protective. The relationship between tau conformation within tau assemblies, its toxicity and role in propagation of pathology are still unclear and the subject of intensive research. Nonetheless, these protein assemblies represent targets for therapeutic strategies and potential biomarkers [10, 14–16, 18].

**Fig. 1:**
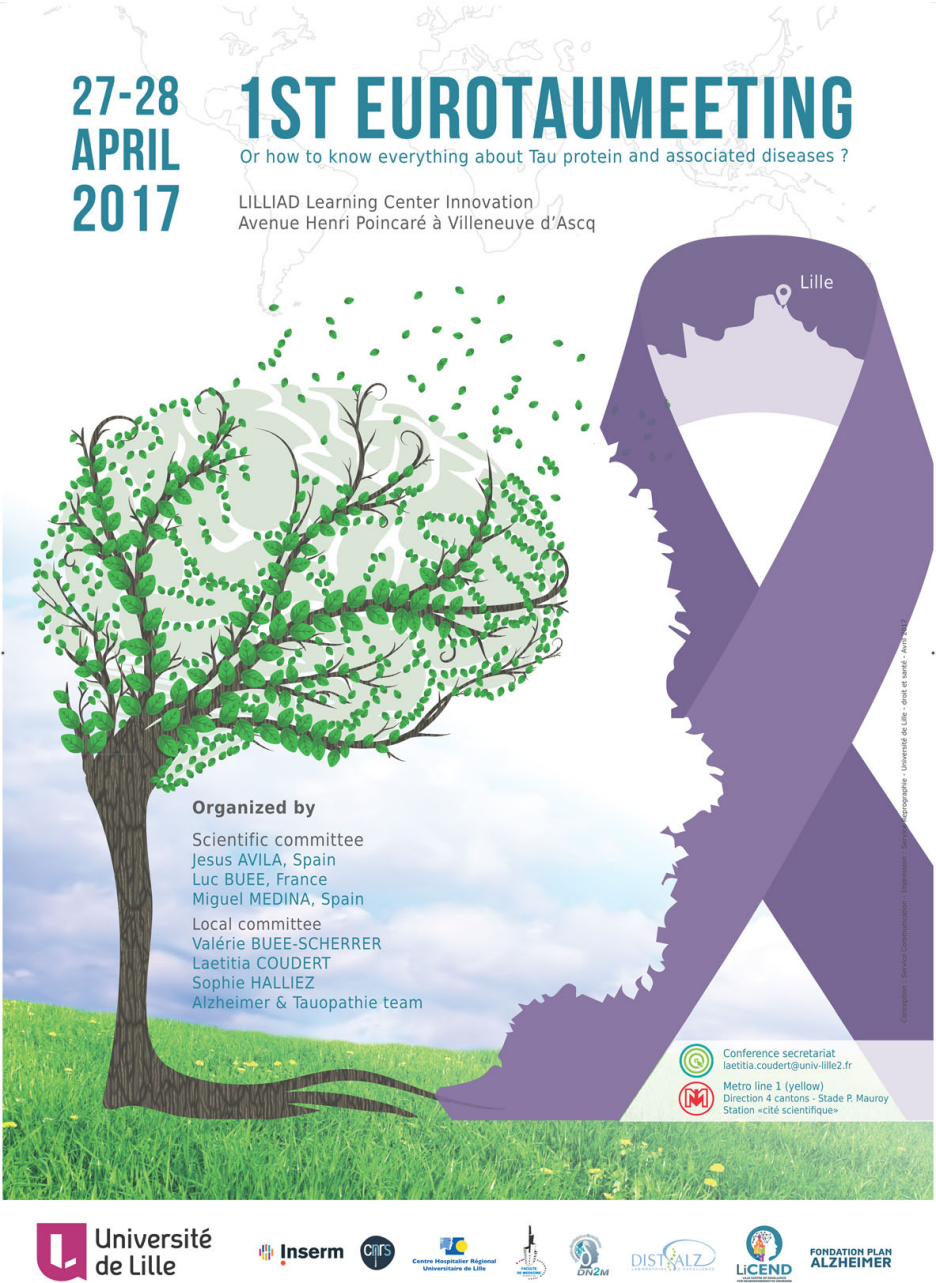
First EuroTau meeting anouncement

In this context, EuroTau is an innovative, collaborative research initiative established to tackle these research questions and the mounting challenges posed by Tauopathies. EuroTau provides a forum for promoting ambitious, innovative, multi-national and multi-disciplinary collaborative research projects in the areas described above, that: i) combine experimental approaches from fundamental, pre-clinical and/or clinical with computational approaches; ii) perform network analyses in different Tauopathies to elucidate the underlying mechanisms that are common as well as those that are different between them, and iii) will add value to existing research by analyzing diseases across traditional clinical boundaries, thereby gaining deeper understanding of the patho-physiological mechanisms of these disorders. The first EuroTau meeting (Scientific Committee: Luc Buee, Miguel Medina, Jesus Avila) was held on April 27–28, 2017 in Lille, France and attracted more than 200 people (Fig. 1). More than thirty-five talks and numerous poster presentations were presented. European funding bodies were also present (Alzheimer Research UK and AFI/ISOA/LECMA-Vaincre Alzheimer). Young and junior investigators were awarded. This meeting was a resounding success and highlighted the need to establish Tau-focused consortia both within Europe, and perhaps worldwide. One key outcome was the instigation of regular such meetings (the next EuroTau meeting will be held on the 26th and 27th of April 2018 in Lille, France. (further details for this meeting can be found at http://lucbuee.fr/crbst_10.html). The other key outcome was the publication of the key ideas discussed as review articles to summarise the discussions, promote ideas for future work and to standardise commonly used but potentially confusing nomenclature.

In this issue of Acta Neuropathologica Communications, summaries of key themes constituting two out of three round table discussions are presented. These included:

1. “What is the evidence that the spread of tau pathology occurs via a prion-like mechanism?” chaired by Amrit Mudher and Jean-Pierre Brion.
2. “Atypical tau functions” chaired by Ioannis Sotiropoulos and Marie-Christine Galas.

The aim of the round table discussions was to reflect on the current state of affairs in these key areas of tau Biology and to make recommendations for future studies. The report of the third round table is also available [7]. Additionally, a talk given by Prof. Maria Spillantini entitled: “Astrocytes in mouse models of tauopathies acquire early deficits and lose neurosupportive functions” was selected for publication as part of this series.

Stay tune and join us at EuroTau 2018 in Lille, France.
